# Reward Uncertainty and Expected Value Enhance Generalization of Episodic Memory

**DOI:** 10.3390/ijerph192114389

**Published:** 2022-11-03

**Authors:** Yang Yue, Yingjie Jiang, Fan Zhou, Yuantao Jiang, Yiting Long, Kaiyu Wang

**Affiliations:** School of Psychology, Northeast Normal University, Changchun 130024, China

**Keywords:** generalization of episodic memory, reward conditioning, consolidation, uncertainty, expected value

## Abstract

Previous research has revealed some mechanisms underlying the generalization of reward expectation of generalization stimuli, but little is known about the generalization of episodic memory for rewarding events, its consolidation, and how reward components such as expected value and reward uncertainty affect it. Participants underwent a Pavlovian reward-conditioning task to test whether reward conditioning would enhance episodic memory generalization and which reward components would directly affect it. Counterbalanced across participants, one semantic category was paired with a reward, while the other was never paired. Following a delay of either 5 min or 24 h, participants took a memory test consisting of old, highly similar, and new items. We found that participants were more likely to falsely recognize lure items as old in the reward-paired category after 5 min and 24 h delays. These results indicate that reward conditioning enhanced the generalization of episodic memory, but this effect was not necessarily dependent on consolidation. The composite score and raw data of generalization further showed that the uncertainty and expected value enhanced generalization. Together, these findings revealed an effect of reward conditioning on episodic memory generalization and supported the enhancement effects of expected value and uncertainty.

## 1. Introduction

Empirically, people are more likely to trust strangers who implicitly resemble those known to be trustworthy than other strangers in the absence of any direct knowledge [1]. This phenomenon whereby past experience can be applied to novel settings is called generalization [2]. With this phenomenon, a question was raised about how generalization happens. Firstly, is the existence of Pavlovian conditioning. It refers to a learning process in which neutral conditional stimuli (CS, such as animals or objects) acquire the capacity to elicit learned conditional responses (CR) by binding with a biologically salient unconditional stimulus (US, such as money) multiple times [3]. Secondly, people can utilize the similarity or relationship between past experiences and novel situations to make an appropriate generalization. For example, previous research has shown that generalization of reward-conditioned responses occurs between two stimuli with similar perceptual or conceptual properties [4,5]. Thirdly, one cannot ignore that memories also form during rewarding experiences and will likely contribute to future discrimination or generalization of similar rewards. However, studies investigating the interplay between episodic memory and stimulus generalization remain rather scarce, leaving a critical question answered. How are the rewarded experiences encoded, and what role do these memories play during generalization? Here, we bridge episodic memory and generalization and try to answer this question by investigating how reward experience affects the generalization of episodic memory. Understanding this question will ultimately shed light on how previously rewarded experiences influence decisions and actions in the present.

Research that directly integrates episodic memory with stimulus generalization remains rare because the generalized conditioned response always entails increased response vigor or choice bias rather than declarative memory enhancement [6]. It is widely known that stimulus generalization occurs between similar perceptual or conceptual stimuli (e.g., generalizing reward expectation from a 300 Hz tone to a 350 Hz tone). In these paradigms, for example, an orientation of a line is associated with a reward (CS+), while a different orientation is associated with no reward (CS−). During the generalization test, participants are presented with a range of lines that vary along different orientations. They not only prefer to respond to test stimuli similar to the CS+, even though these stimuli have not been directly associated with reward, but also respond more to a value beyond the CS+ in the direction away from CS−. This phenomenon is called “peak-shift”, which is found in animals such as pigeons or rats [7] and in human stimulus generalization research [5], although some research has demonstrated that the hippocampus, a physiological structure supporting declarative memory, plays a crucial role in stimulus generalization [4]. Most research on stimulus generalization has predominantly been studied independently with episodic memory.

However, it is necessary to examine memory in stimulus-generalization research because episodic memory formed during the reward conditioning phase will affect the identification (or misidentification) of similar rewards to a great extent. For example, if a person more clearly remembers that a golden retriever with long hair was paired with a reward, they are less likely to make a reward prediction with a labrador retriever with short hair. Fortunately, a few studies initiated this investigation, thus providing an initial idea of the underlying dynamics between memory and stimulus generalization. An example of this was demonstrated by Starita and colleagues in 2019 [8], who showed that aversive conditioning could promote episodic memory generalization. In their study, pictures from one category were paired with an electric shock 50% of the time, while the objects of a different category were unpaired during Pavlovian threat conditioning. The following day, a recognition memory test was administered based on paradigms used to evaluate behavior pattern completion and separation. Participants needed to identify whether an item was “old”, “similar”, or “new”, which included old targets (i.e., the picture on the screen was exactly like a picture seen on Day 1), highly similar lures (i.e., the picture on the screen was different from, but similar to, a picture seen on Day 1, e.g., during acquisition a dog was presented and during the memory test the same dog but with different body position was presented), and entirely novel foils (i.e., the picture on the screen was a unique exemplar of an object that was never presented on Day 1) from the shock-paired and unpaired categories. The result showed that, compared to lures from unpaired categories, lures in the threat-conditioned category were more likely to be endorsed “old”. Therefore, we could adopt the same approach to investigate whether reward conditioning can promote the generalization of episodic memory.

Our second goal is to investigate whether episodic memory generalization is consolidation-dependent. Previous studies have shown that reward improves episodic memory by promoting memory consolidation [9]. This idea is commonly tested by measuring memory immediately after learning or at delayed time points [10]. As a result, compared with a memory test carried out immediately after the learning session, the memory difference between high and low rewards is usually greater after a 24 h delay, and the period of 24 h after encoding is bound to involve sleep. This pattern likely reflects at least two important neuro-processes; (i) the hippocampus’s replay of memory traces, especially during sleep on the first night after learning [11,12], and (ii) the fact that dopamine can affect hippocampal plasticity and memory on a longer timescale after events are experienced [13]. Thus, the 24 h delay test possibly allows reward-conditioned items sufficient time to be well consolidated and consequently better remembered than the unrewarded conditioned stimuli. Given that Starita and colleagues in 2019 only investigated the effect of aversive conditioning on episodic memory generalization under the 24 h delay test, the question as to whether the effect of reward conditioning on episodic memory generalization is consolidation-dependent remains answered. For this reason, the present study compared the differences in memory generalization between 5 min and 24 h delay test conditions.

The third purpose of the current study is to clarify which reward components can predict episodic memory generalization. According to previous research on single-cell neurophysiology, the dopaminergic reward system responds promptly to different reward components, namely, expected value, outcome, uncertainty, and reward prediction error (RPEs) [14,15], leading one to presume that different components may have different effects on generalization. This is especially true for the reward prediction error, which is involved in the generalization process of reward conditioning in stimulus generalization [4]. For example, Kahnt et al. in 2012 found that functional connections between the hippocampus and the ventral striatum controlled dopamine transmission induced by prediction error and regulated the width of generalization. In the same vein, studies also showed that uncertainty served as a potent stressor that induced fear stimulus generalization [16]. Overall, it is unclear whether such enhanced effects of reward conditioning are driven by RPEs or uncertainty rather than other reward components of episodic memory generalization. Our study set out to explore and answer this question.

In short, the present study investigates the effect of reward conditioning on the generalization of episodic memory and examines which components of the reward signal can predict it. Three main hypotheses are tested. First, whether reward conditioning enhances memory generalization. Second, whether the impact of reward on generalization depends on memory consolidation, indexed by a more robust consolidation in the 24 h delay test condition compared to the 5 min delay test (both hypotheses tested in Experiment 1). Third, we assess the extent (if any) to which reward components such as reward prediction error and uncertainty can predict the reward effect on generalization (Experiments 2a and 2b).

## 2. Experiment 1

Experiment 1 examines whether reward conditioning promotes episodic memory generalization and whether the enhancement is consolidation-dependent. We used reward conditioning as the within-subject factor and time interval as the between-subject factor. The former featured two conditions: reward-paired (CS+) and reward-unpaired (CS−). The latter included two time-interval conditions: 5 min and 24 h delays. We calculated the participants’ corrected memory score (responding “old to targets” minus responding “old to foils”), episodic memory generalization score (responding “old to lures” minus responding “old to foils”) as the dependent variables according to the methodology adopted in Starita in 2019 [8].

### 2.1. Materials and Methods

#### 2.1.1. Participants

Our sample size was determined through a power analysis using G*Power software [17]. The sample size needs to achieve a medium effect size (*f* = 0.25) to detect a within-between interaction with the repeated measures ANOVA. We calculated it using 0.05 as an alpha error probability, 95% as power, 0.50 as a correlation among repeat measures level, and 1 as the non-sphericity correction e. The result showed a 95% chance of correctly rejecting the null hypothesis of no significant interaction effect with 27 in the 5 min delay test group and 27 participants in the 24 h delay test group. A total of 81 participants were recruited from the local university in return for monetary compensation in the local currency, the Chinese yuan (CNY). All participants had normal or corrected-to-normal vision and were unaware of the experiment’s purpose. They gave written consent prior to the experiment. Forty-four participants were assigned to the 24 h delay test group. The exclusion criteria for the 24 h delay test group were as follows: participants who failed to finish day 1 (*n* = 3), failed to return for day 2 (*n* = 3), correct recognition memory lower than 0.1 (*n* = 1, which meant failure to demonstrate recognition memory above chance) and false alarm rate exceeding 70% (*n* = 2, which meant that participants did not pay enough attention in the reward conditioning phase). In the end, data from 35 participants were analyzed (8 males; age *M* = 21.43 years, *SD* = 2.06 years). Thirty-seven participants were assigned to the 5 min delay test group. Participants who failed to finish the conditioning phase (*n* = 1) and exceeded 70% of the false alarm rate (*n* = 3) were removed from the analysis. Finally, the data of 33 participants were analyzed (8 males; age *M* = 20.84 years, *SD* = 1.83 years). 

#### 2.1.2. Materials

Pictures were collected from Oyarzún’s article [18], Mnemonic Similarity Task created by Craig Stark [19], the website http://www.lifeonwhite.com (accessed on 28 October 2022), and from publicly available resources on the Internet. In total, sixty animal and sixty object pictures were used as trial stimuli in the incidental encoding task, and two animal and two object pictures were used as practice. We did not present stimuli of the “same basic level” (e.g., two different pictures of a cat). In the surprise recognition memory task, the participants were shown random stimuli that included 60 images used as old targets (precisely the same photos used in the conditioning phase), 60 images used as similar lures (the same animal or the same object used in the conditioning phase but viewed in the test phase in different positions and angles, respectively), and 60 images used as novel foils (pictures never presented in the conditioning phase, 30 animal and 30 object pictures). The number of old targets, similar lures and novel foils was equally divided into the reward-paired (CS+) and reward-unpaired (CS−) categories.

#### 2.1.3. Procedure

**Figure 1:**
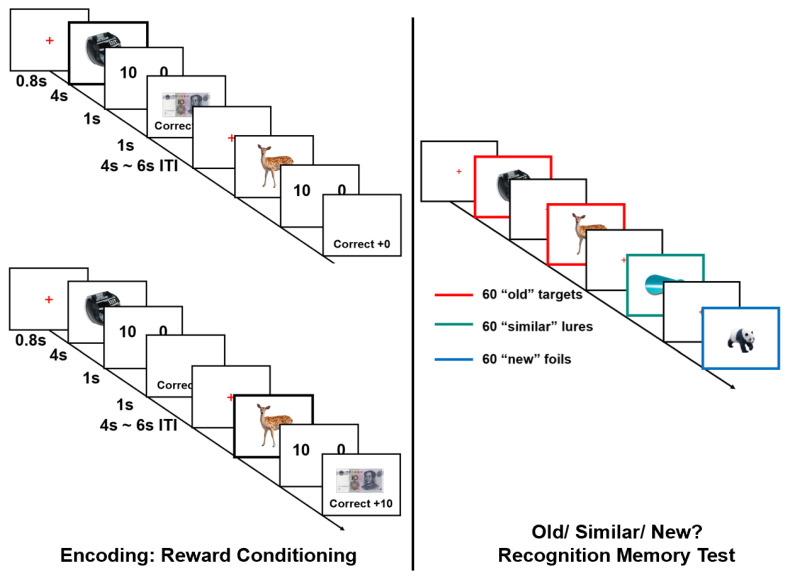
In the incidental encoding task, participants encode pictures incidentally by completing a value-guessing task. In the recognition memory test, participants were asked to identify whether the picture was old, similar, or new. Bold and colored borders were used only for illustration purposes and were not part of the stimuli shown to participants.

The procedure was similar to that used in Starita in 2019 with two experimental sessions: an incidental encoding task and a surprise recognition memory test (see Figure 1).

The incidental encoding task was a reward learning task with pre-deterministic associations between picture category and value, which included the picture presentation, the reward expectancy, and the feedback phase. Sixty color pictures of animals and 60 color pictures of objects were presented on a white background. For the reward-conditioned category (e.g., CS+, animals or objects counterbalanced), 30 pictures were rewarded with CNY 10 (we determined which trial was accomplished with CNY 10 in the CS+ category by generating random numbers in Excel), whereas the remaining 30 pictures terminated with CNY 0. A within-subject control category (e.g., CS−, the alternative category) was never paired with a reward; all 60 trials ended with CNY 0. The object categories working as CS+/CS− were counterbalanced between subjects, and each image was presented just once. During the task, each picture was presented for 4 s with an inter-trial interval of 5 ± 1 s. It allowed enough time to decrease the impact of the feedback from the former trial on the next trial. After each picture disappeared, participants were asked to indicate whether the image would receive CNY 10 or 0 by pressing the keyboard keys “F” or “J”, respectively. They should respond in 1 s to ensure they concentrate on the task. Then, if their guesses were correct, they would receive the corresponding value and be shown “Correct” feedback on the screen. If participants’ guesses were wrong, they would lose CNY 1 and receive “Wrong, −1CNY” feedback as a form of warning to improve task performance in the subsequent trial. They learned the category-value associations through the reward feedback with multiple experiences instead of being instructed about the conditioned–unconditioned stimulus contingencies. Participants were advised to pay close attention to the feedback of pictures to learn the association of reward and category and correctly decide the reward value of the following picture to get higher accumulated points. The total points were converted into compensation fees of 20 to 1 at the end of the task. After the incidental encoding task, a manipulation check was conducted by asking participants, “Did you find the corresponding relationship between category and reward value?” All subjects gave the right answer accordingly.

The surprise recognition memory task was similar to a Mnemonic Similarity Task using image stimuli that were presented during the learning task, similar lures, and new foils. It was performed at two different time intervals. One group performed the memory test after 5 min, while the other group took the test after 24 h. To prevent participants from knowing about the follow-up memory test, they were informed that the same task with different pictures would be conducted in the second block later (for the 5 min delay test group) or the next day (for the 24 h delay test group) [18]. During the test, participants were presented with animal and object pictures; they should respond with “Left”, “Up”, and “Right” arrows on each trial to report whether the item was an “old target”, “similar lure”, or “new foil”, respectively. The memory test was self-paced to obtain all the data of the subjects and remove their anxiety about limited time. A jittered 800 ms inter-trial interval followed each trial. No feedback was provided during the test.

#### 2.1.4. Data Analysis

We used the corrected memory score as the recognition memory performance and used the generalization score as the episodic memory generalization. Therefore, for each score, the mixed-measure ANOVA with time interval (5 min delay vs. 24 h delay) as a between-subject factor and reward conditioning (CS+ vs. CS−) as a within-subject factor was performed, respectively.

According to the definition of the reward component in Table 1, we could calculate the reward-related predictors in each experiment. First, for Experiment 1, the probability of obtaining a reward was preset (50% vs. 0%). Therefore, a higher expected value (10 × 50% = 5) was bound to the high reward category. The outcome was defined as the predetermined value of the picture that the participant could learn from feedback (e.g., CNY 10 and 0). Therefore, we could calculate SRPEs and URPEs at the feedback phase by taking the value or absolute value of the difference between the expected value and the reward outcome, such as Rouhani in 2021 [20]. In addition, a common measure of uncertainty is entropy. Entropy is calculated as minus the weighted sum of the logarithm of the probabilities of each possible outcome. It follows an inverted U-shaped function of the reward probability and is minimal at *p* = 0 and *p* = 1 and maximal at *p* = 0.5. For example, when the reward occurs for sure (*p* = 1), the uncertainty is minimum as 0; however, when the reward occurs with a 50% probability, the uncertainty is maximum as 1 [15].

### 2.2. Results and Discussion

We present the response proportions for each stimulus and response type in Table 2.

#### 2.2.1. Effects of Reward Conditioning and Time Interval on Stimulus Recognition

**Figure 2:**
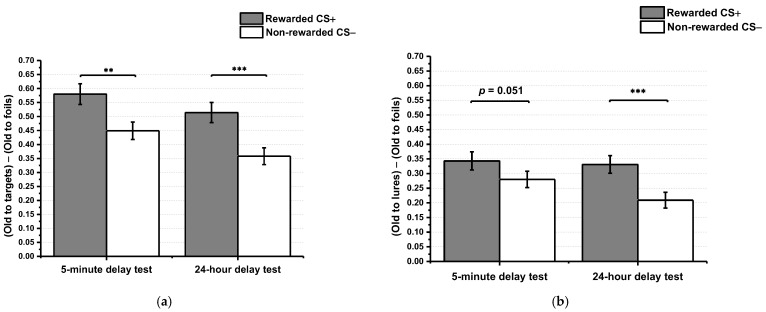
(**a**) Recognition memory performance. The corrected memory score was greater for items in the CS+ compared to the CS− category in the 5 min delay test condition and the 24 h delay test condition. For example, the mean corrected memory score of the CS+ category in the immediate test condition was 0.59, obtained by 0.66 (the mean of responding old to targets) minus 0.07 (the mean of responding old to foils) in the first row of Table 2. (**b**) Episodic memory generalization. The episodic generalization score was greater for items in the CS+ compared to the CS− category in the 5 min delay test condition and the 24 h delay test condition. For example, the mean generalization score of the CS+ category in the 5 min delay test condition was 0.34, obtained by 0.41 (the mean of responding old to lures) minus 0.07 (the mean of responding old to foils) in the first row of Table 2. Error bars represent ± *SEM.* *** *p* < 0.001, ** *p* < 0.01.

For the corrected memory score, the main effect of conditioning was significant, *F* (1, 66) = 21.21, *p* < 0.001, *η*_p_^2^ = 0.24. Participants showed an increase in corrected memory score of CS+ (*M* = 0.55, *SE* = 0.03) compared with CS− (*M* = 0.40, *SE* = 0.02). The main effect of time interval was also significant, *F* (1, 66) = 4.63, *p* = 0.035, *η*_p_^2^ = 0.07. Participants had significantly higher corrected memory scores in the 5 min delay test condition (*M* = 0.52, *SE* = 0.03) than in the 24 h delay test condition (*M* = 0.44, *SD* = 0.03). The interaction was not significant, *F* (1, 66) = 0.17, *p* = 0.679, *η*_p_^2^ = 0.01 (See Figure 2a). The extant results demonstrated that items in the reward-paired category were better remembered than items in the category never paired with a reward, whether the memory test was delayed by 24 h or only 5 min.

#### 2.2.2. Effects of Reward Conditioning and Time Interval on Episodic Memory Generalization

For the episodic memory generalization score, in line with our predictions, the main effect of conditioning was observed, *F* (1, 66) = 17.53, *p* < 0.001, *η*_p_^2^ = 0.21. Post hoc analysis revealed that the generalization score in the CS+ category (*M* = 0.34, *SE* = 0.02) was significantly higher than that in the CS− category (*M* = 0.24, *SE* = 0.02). The main effect of the time interval was not significant, *F* (1, 66) = 1.45, *p* = 0.233, *η*_p_^2^ = 0.02. Likewise, the interaction was not significant, *F* (1, 66) = 1.77, *p* = 0.188, *η*_p_^2^ = 0.03 (see Figure 2b). Similar to the results reported in the corrected memory score analysis, the lack of the main effect of time interval and interaction does not support the idea that episodic memory generalization is consolidation-dependent. In addition, a memory difference score (corrected generalization score for CS+ items minus CS− items) was used to assess whether the generalization enhancement differed for 5 min delay and 24 h delay test conditions [21]. It revealed that there was no significant difference between the generalization scores in the 5 min delay test group relative to the 24 h delay test group (*t*_66_ = −1.33, *p* = 0.188, two-tailed, *d* = 0.32). Based on that, the evidence supports that the effect of reward conditioning on generalization is not consolidation-dependent.

In sum, we used the incidental encoding and surprise recognition memory test paradigm to investigate how reward conditioning influences the generalization of episodic memory in Experiment 1. We found that reward conditioning enhanced the subsequent generalization of episodic memory, henceforth supporting hypothesis 1. We did not find supporting evidence for hypothesis 2—that the impact of reward conditioning on episodic memory generalization depended on memory consolidation. However, we could not test how reward components influenced episodic memory generalization because of the 100% correlation between the expected value, uncertainty, and URPE in Experiment 1. To address this issue, we made the expected value in each category equal to investigate the effect of uncertainty or URPE on generalization in Experiment 2a while making the uncertainty in each category equal to compare the effects of expected value and URPE in Experiment 2b.

## 3. Experiment 2a

Experiment 2a aimed to control the expected value and investigate the effect of uncertainty or UPRE on generalization. According to Experiment 1, the effect of reward conditioning on episodic memory generalization was more robust in the 24 h delay test condition than in the 5 min delay test condition. Therefore, in Experiment 2, only the 24 h time interval between conditioning and the test was conducted.

Experiment 2a employed a one-way repeated-measures design with uncertainty as a factor with two levels: high uncertainty (which equals 1, by CNY 0 or 10 with equal 50% probability) and low uncertainty (which equals 0, by CNY 5 with 100% reward rate), which made the expected value equal in each category (5, 5). Specifically, in the high uncertainty condition, 15 of 30 pictures were rewarded with CNY 10, whereas the remaining 15 pictures resulted in CNY 0. The picture-reward assignment results in a higher (or lower) than expected value (5) in this condition, which can result in a larger URPE (5) at the reward feedback screen, according to the formula in Table 1. The low uncertainty category consisted of 30 trials, all ending with CNY 5, which matched the expected value (5) of this category resulting in a smaller URPE (0). Consequently, the participants experienced different levels of uncertainty (1, 0) but with the same levels of expected value (5, 5) at the picture presentation screen; however, they experienced different levels of URPEs (5, 0) at the reward feedback screen with different categories of pictures. Although the uncertainty and URPE were 100% correlated in Experiment 2a, people experienced uncertainty first during the picture presentation phase. Consequently, we still define uncertainty as an independent variable. Each category paired with high uncertainty was counterbalanced between participants.

### 3.1. Materials and Methods

#### 3.1.1. Participants

Because the effect size of reward learning on episodic memory generalization in the delayed condition in Experiment 1 (the mixed effect of expected value, uncertainty, and URPE) was 0.68, we estimated the effect size in Experiment 2a (the mixed effect of uncertainty and URPE) to be 0.453 (0.68 divided by 3 is 0.227, 0.227 multiplied by 2 equals 0.453). Therefore, we calculated the sample size with two tails paired *t*-tests, an alpha level of 0.05, 80% power, and a 0.453 effect size. The result showed that there is an 80% chance of correctly rejecting the null hypothesis of no significant difference between the two dependent means in the high and low uncertainty category with a total of 41 participants. In Experiment 2a, 45 participants with normal or corrected-to-normal vision were recruited from the local university for monetary compensation. Participants were removed from the analysis if they failed to finish day 1 (*n* = 1), if corrected recognition memory was lower than 0.1 (*n* = 1), and if the false alarm rate exceeded 70% (*n* = 1). The data of 42 participants were analyzed (10 males; age *M* = 21.73 years, *SD* = 3.29 years).

#### 3.1.2. Procedure

The material and procedures used in the current experiment were similar to the 24 h delay condition of Experiment 1. In Experiment 2a, after the participants saw the pictures, they were required to report whether they expected to receive CNY 10, 5 or 0 by pressing the keyboard keys “A”, “W”, or “D”, respectively. Other settings were consistent with Experiment 1.

#### 3.1.3. Data Analysis

For the composite scores, to detect the effect of uncertainty on the generalization score, a paired sample *t*-test on corrected memory score and episodic memory generalization score was conducted in Experiment 2a first. In addition, for Experiments 2a and 2b, we also performed two types of generalized linear mixed models on individual trials in R [22]. The trial-by-trial analysis could test which reward components could predict it and assess the unique contribution of predictors (i.e., expected value, outcome, uncertainty, signed reward prediction error, and unsigned reward prediction error. Table 1 shows the definitions of the five predictors. We defined the variables as categorical because they only contained a few levels, not continuous). The dependent variable of regression was “0” or “1”, representing subjects’ response to lure pictures separately shown in the test phase. For example, when a participant reported “old” to a lure, it was coded as “1” in the generalization column (other responses were coded as 0). The first type of regression contains three kinds of responses (responding old to lures, responding similar to lures, and responding new to lures) to test which reward components helped participants to make more generalization responses. For the second one, we deleted new responses and only contained two types of reactions to check which reward components led participants to make a generalization response and which ones led to a discrimination response when they realized they had seen the picture before. There are two benefits of this approach. The first one is that it allowed us to consider individual differences through a random effect and test the unique contribution of predictors. The second one is that we could compare the results from the two types of regression induced by different data classifications further to investigate the boundary of the reward enhancement effect.

We combined a series of methods in the process of modeling to find the best fit model. Firstly, we used the “findLinearCombos” function in the “caret” package to enumerate and resolve the linear combinations in a matrix [23], used the “glm” function in the “lme4” package to construct the full model, and checked the significance of fixed factors [24]. Secondly, we then used the “stepAIC” function with a backward direction in the “MASS” package to simplify the model and retain the significant fixed effect factors [25] and used the “glmer” function in the “lme4” package to explore the random effects [24]. At the same time, the Bayesian Information Criteria (BIC) of models were reported. Thirdly, we selected the model with the lowest BIC value and then compared the other models’ BIC values to this model. We calculated the Bayes Factor (*BF*), which can be converted to an approximation according to the following rule: *BF_(_*_M1 − M2)_ = exp (−0.5 × (*BIC*_M1_ − *BIC*_M2_) [15]. By convention, it is suggested that odds greater than three represent “some” evidence for a model over another, whereas odds greater than ten represent strong evidence.

### 3.2. Results and Discussion

#### 3.2.1. Effect of Reward Uncertainty on Stimulus Recognition and Episodic Memory Generalization

Descriptive statistics of the rate of old/similar/new responses for each stimulus type in Experiments 2a and 2b are reported in Table 2. In Experiment 2a, a paired sample *t*-test revealed a significantly higher corrected memory score for the high uncertainty category (*M* = 0.48, *SE* = 0.03) than the low one (*M* = 0.38, *SE* = 0.03), *t* (41) = 2.61, *p* = 0.013, *d* = 0.40, 95% CI [0.02, 0.19]. Likewise, the analysis of the generalization scores also revealed a significantly greater generalization for items of the high uncertainty (*M* = 0.33, *SE* = 0.02) than the low category (*M* = 0.21, *SE* = 0.02), *t* (41) = 3.92, *p* < 0.001, *d* = 0.60, 95% CI [0.03, 0.06]. These results indicate that uncertainty could enhance item recognition and episodic memory generalization.

#### 3.2.2. Trial-by-Trial Regression Analysis on Generalization

For the generalized linear regression with three kinds of response, there was a significant difference in generalization for uncertainty (*β* = 0.56, *SE* = 0.08, *z* = 6.72, *p* < 0.001) but not for the outcome (*β* = 0.01, *SE* = 0.01, z =1.29, *p* = 0.197). When considering individual differences in the generalized linear mixed model, the evidence showed that uncertainty was an efficient, positive predictor of generalization (*β* = 0.59, *SE* = 0.14, *z* = 4.17, *p* < 0.001). Moreover, it could serve as a random slope at the individual level in the random effect (σ^2^ = 0.51), indicating that uncertainty could partially explain individual differences (See Table 3). The result showed that reward uncertainty promotes individuals to make more generalization responses in lures.

For the generalized linear regression with only generalization and discrimination responses, similarly, there was a significant difference in generalization for uncertainty (*β* = 0.31, *SE* = 0.10, *z* = 2.98, *p* = 0.003) but not for the outcome (*β* = 0.01, *SE* = 0.01, *z* = 0.95, *p* = 0.340). However, when considering individual differences in the generalized linear mixed model, we found that the final model, which had the smallest BIC, showed the effect of uncertainty on generalization as weak or even insignificant (*β* = 0.28, *SE* = 0.22, *z* = 1.27, *p* = 0.204). Therefore, Experiment 2a showed that the reward uncertainty at the encoding phase could help individuals recognize the item as old in the memory test. However, it may have a limited effect on generalizing or discriminating when they realize they have seen the item before.

Experiment 2a replicated the finding from Experiment 1 that pairing a category with a higher reward uncertainty could improve episodic memory generalization. In addition, we found the strongest evidence in favor of a model that the reward uncertainty worked as the fixed effect and random effect in the data of three kinds of response. But when only retaining the old and similar response to lures, which means participants believed they had seen the item in the reward conditioning phase, they may not depend on the uncertainty to make the generalization or discrimination decision. Therefore, the result indicated that the uncertainty experienced in the encoding phase could help individuals remember and recognize the item as they have seen it. However, it may have a limited effect on helping people generalize or discriminate between similar lures and old targets.

## 4. Experiment 2b

Experiment 2b aimed to control uncertainty to examine the effect of expected value and URPE on generalization. It employed a one-way repeated measures design with expected value as a factor with two levels: high expected value (CNY 10 with 80% reward rate and CNY 0 with 20% reward rate) and low expected value (CNY 10 with 20% reward rate and CNY 0 with 80% reward rate), which made the uncertainty (0.22, 0.22) equal in each category. Specifically, twenty-four pictures were rewarded with CNY 10 for the high expected value category, higher than the expected value (8), causing a smaller URPE (2) at the reward feedback screen. The remaining six pictures were rewarded with CNY 0, which is lower than this category’s expected value (8), causing a larger URPE (8) at the reward feedback screen. The average URPE of each trial was 3.2. Although the URPE’s calculation of the low expected value category is opposite to the high expected value category, the calculation result of URPE is the same (3.2). Therefore, participants experienced the same levels of uncertainty (0.22, 0.22) but different levels of expected value (8, 2) at the picture presentation screen and experienced the same levels of URPEs (3.2, 3.2) at the reward feedback screen with different categories of pictures. Each category paired with a high expected value was counterbalanced between participants.

### 4.1. Materials and Methods

#### 4.1.1. Participants

To be comparable to the subject number of Experiment 2a, we recruited 57 participants with a normal or corrected-to-normal vision from the local university for monetary compensation. Participants were removed from the analysis if they failed to return for day 2 (*n* = 3) and if the corrected recognition memory was lower than 0.1 (*n* = 2). The data of 52 participants were analyzed (8 males; age *M* = 20.81 years, *SD* = 1.50 years).

#### 4.1.2. Procedure

This experiment had the same settings as Experiment 1, except that one category received 80% of the CNY 10 reward while the other received 20%.

#### 4.1.3. Data analysis

Similar to Experiment 2a.

### 4.2. Results and Discussion

#### 4.2.1. Effect of Expected Value on Stimulus Recognition and Episodic Memory Generalization

In Experiment 2b, a paired *t*-test revealed a significantly greater corrected memory score for items of the high expected value category (*M* = 0.50, *SE* = 0.03) than the low expected value category (*M* = 0.42, *SE* = 0.02), *t* (51) = 2.08, *p* = 0.043, *d* = 0.29, 95% CI [0.00, 0.16], and also revealed a significantly higher generalization score for items of the high expected value category (*M* = 0.32, *SE* = 0.02) than the low category (*M* = 0.24, *SE* = 0.02), *t* (51) = 2.77, *p* = 0.008, *d* = 0.38, 95% CI [0.02, 0.14]. The above results suggest that the expected value could enhance item recognition and episodic memory generalization.

#### 4.2.2. Trial-by-Trial Regression Analysis on Generalization

For the generalized linear regression with three kinds of response, there was a significant difference in generalization for expected value (*β* = 0.07, *SE* = 0.02, *z* = 4.31, *p* < 0.001) and a marginally significant difference for URPE (*β* = −0.03, *SE* = 0.02, *z* = −1.72, *p* = 0.084), but not for the outcome (*β* = 0.001, *SE* = 0.01, z = 0.15, *p* = 0.883). Therefore, we adopted the expected value and URPE into the generalized linear mixed model. When considering individual differences, the model comparison result showed that only the expected value was an efficient, positive predictor of generalization (*β* = 0.46, *SE* = 0.13, *z* = 3.45, *p* < 0.001). Moreover, it could serve as a random slope at the individual level in the random effect (σ^2^ = 0.59), indicating that the expected value could partially explain individual differences (See Table 4). The result showed that only expected value promotes individuals to make more generalization responses in lures.

For the generalized linear regression only with generalization and discrimination responses, similarly, there was a significant difference in generalization for expected value (*β* = 0.05, *SE* = 0.02, *z* = 2.75, *p* = 0.006), and URPE (*β* = −0.05, *SE* = 0.02, *z* = −2.96, *p* = 0.003), but not for the outcome (*β* = 0.01, *SE* = 0.01, *z* = 0.65, *p* = 0.515). When considering individual differences in the generalized linear mixed model, we found that the final model, which had the smallest BIC, showed that the negative effect of URPE on generalization was significant (*β* = −0.41, *SE* = 0.12, *z* = −3.36, *p* < 0.001), and the positive effect of expected value was marginally significant (*β* = 0.33, *SE* = 0.18, *z* = 1.83, *p* = 0.067). Therefore, Experiment 2b showed that the expected value at the encoding phase could help individuals recognize the item as old in the memory test. However, it may have a weak effect on generalizing or discriminating when they realize they have seen the item before. But the URPE at the feedback phase has a powerful role in helping people discriminate between lures and targets, decreasing generalization.

Integrating the results of the composite score and raw data of generalization, Experiment 2b provides evidence that the expected value enhanced; however, the URPE decreased the generalization of episodic memory. By comparing the results of the two kinds of regression analysis, we further found that the expected value in the picture presentation phase could help individuals remember the item and recognize it as old. However, it may have a limited effect on generalizing or discriminating between similar lures. The URPE could help people discriminate between the lures and targets and decrease generalization.

## 5. Discussion

Episodic memory formed during rewarding experiences will help people guide value-based adaptive decisions in similar future situations. The present study aimed to examine the effect of Pavlovian reward conditioning on long-term episodic memory generalization by testing whether this effect was consolidation-dependent and by exploring which reward component predicted it. We found that reward conditioning not only enhanced item recognition but also promoted episodic memory generalization. Nevertheless, the enhancement of reward conditioning on generalization was not necessarily dependent on sleep consolidation. We also found that uncertainty and the expected value of reward enhanced long-term episodic memory generalization. In contrast, unsigned reward prediction error decreased it.

### 5.1. Reward Conditioning Promotes Episodic Memory Generalization

The results presented herein consistently showed that reward conditioning promoted episodic memory generalization, in accordance with a recent study that employed a threat-conditioning paradigm [8]. Furthermore, our results are consistent with a reward-spreading effect based on perceptual or conceptual similarity, where reward could incidentally enhance neutral memories related to rewarded items. For example, previous research has shown that the memory of similar lures that surprisingly appeared within serial repetitions of a trial-unique, color object image could be enhanced by reward motivation [26]. In the same way, a weak memory of similar conceptual items could be improved by a former reward-conditioned experience [18,27]. Our results also consist of a reward transfer effect based on the same outcome or episode [28,29]. According to the tag-and-capture hypothesis, dopamine strongly affects late long-term potentiation (LTP). This view suggests that a “penumbra” surrounding event causes dopamine release; memory for items related to previously rewarded items at a conceptual level or events that occur before or after the dopamine release is enhanced [30].

We also found that consolidation processing is not necessary for the effect of reward conditioning on episodic memory generalization. We propose that different types of generalization may not be equally affected by consolidation during sleep [31]. Precisely, the similarity between the conditioned and the generalization stimuli may determine whether the reward effect on generalization is consolidation-dependent. If the conditioned and the generalization stimuli are represented in the brain separately (as in an acquired equivalence task), the “offline” state at rest or overnight sleep after conditioning is helpful to integrate or bridge learning experiences together for generalizing or inferencing [32]. In addition, earlier studies have also proposed a crucial role of sleep in generalization [2,31,33]. Conversely, when the difference between conditioned and generalization stimuli is minimal, if the original representation of the conditioned stimuli can be recollected, the representation of the generalization stimuli can also be activated; hence, the consolidation process is not necessary. It is plausible that the similarity between conditioned and generalization stimuli may be the boundary condition of the reward effect. This topic, however, namely, memory consolidation and reward interaction in episodic memory generalization, is an interesting question for further investigation.

### 5.2. Uncertainty and Expected Value Are Positive Predictors of the Generalization of Episodic Memory

According to the regression results, reward uncertainty and the expected value were positive predictors for the generalization of episodic memory. We supposed that the reward anticipation caused by uncertainty and expected value might enhance the gist memory of items, enhancing the generalization of episodic memory observed in our results. One explanation for lure false alarms is the fuzzy-trace theory (FTT) of false memory [34]. It proposes that events are encoded in two kinds of traces: verbatim and gist trace. Verbatim traces are defined by a mental reinstatement of the target event (the event’s surface perceptual forms), helping decrease false memory. However, gist traces are thought to represent an event’s semantic features (the meaning of an event), which can increase false memory. Since tasks assessing discrimination and generalization of episodic memory adopt lures that share gist traces with studied items, the availability of verbatim traces in memory would help the successful rejection of lures. In contrast, the availability of gist traces would help recognize the lures as old items, enhancing episodic memory generalization.

Moreover, previous studies have shown that reward anticipation or motivation can promote gist memory [35,36]. For instance, people have a reward-related memory benefit specific to objects from pairs encoded in the attention-to-gist condition but not in the attention-to-detail condition, meaning that reward anticipation selectively boosted the encoding of gist for objects [36]. This result supports the idea that reward in associative generalization can be transferred by operating on a meaningful or abstract level of representation. In addition, people also could generalize memory to make adaptive decisions about a face on a higher, view-invariant level of abstraction in an acquired equivalence task [37]. In theoretical and neuroimaging research, as the hypothesis of the long-axis specialization of the human hippocampus, gist-based memory representations rely on the anterior hippocampus, whereas detailed-based memory representations depend on the posterior hippocampus [38]. It is well established that anticipation of reward or novelty can activate mesolimbic regions, which directly project to the anterior hippocampus [39,40]. Therefore, the whole map of results above supported that reward anticipation could enhance the gist trace of memory.

As for the effect of uncertainty and expected value investigated in our research, we propose that both can trigger reward anticipation to enhance the gist trace, therefore improving the generalization of episodic memory. Although some researchers have shown that expected reward and uncertainty have spatial and temporal neutral differentiation in human subcortical structures, for example, initial activation in the ventral striatum and other subcortical dopaminoceptive structures varied with expected reward, whereas subsequent activation in the ventral striatum varied with uncertainty [41], they still have some properties in common that can trigger the anticipation of reward before the outcome is delivered. Moreover, neuroimaging studies provide relevant evidence that the ventral tegmental area, which can be activated by expected value, and the insula, which can be activated by uncertainty, both have long-range connections to the human anterior hippocampus [38,41]. Consequently, we propose that the expected value and uncertainty of reward can enhance the gist trace and then enhance the generalization of lures. The imbalance of gist and verbatim trace enhancement may be the primary cause of the enhancement effect of reward conditioning on generalization. Nonetheless, further studies are needed to verify this possibility.

### 5.3. Unsigned Reward Prediction Error Worked as a Negative Predictor of Generalization of Episodic Memory

Our results showed that an unsigned reward prediction error negatively affected episodic memory generalization. An item with a larger URPE helped people distinguish between the recognition lures and previous encoded targets, thus reducing generalization. The result is consistent with previous studies on stimulus generalization [4]. We also proved that memory specificity performed after reward conditioning likely affects how we generalize from prior rewarded experience. Hence, we propose an intriguing possibility that reward prediction errors may have enhanced the salience of items, thus helping subjects create a distinct memory representation, reducing interference from lures, and supporting its correct rejection. Recent studies on prediction error modulation of memory align with this idea [20,42,43]. For instance, studies showed that unsigned reward prediction error reduces interference from prior memories by creating a more distinct memory trace. High RPE enhanced the item recognition but worsened sequence memory for pairs, reflecting the interference and relation between items across a high-RPE event [42]. Another well-known type of prediction error, mnemonic prediction errors, which can be induced by a mismatch between predicted and received information, can also enhance memory by facilitating detailed, high-fidelity memory, potentially reflecting distinct memory traces [44]. In short, a high level of URPE may help people create a distinctive memory trace and decrease episodic memory generalization. Future work could further check how URPE strengthens mnemonic details, reduces interference, and decreases the generalization of episodic memory.

## 6. Conclusions

To summarize, we conclude that reward conditioning can promote episodic memory generalization, an effect that is not necessarily dependent on consolidation. Further, we also demonstrated that reward uncertainty and expected value enhanced the long-term generalization of episodic memory. These results extend findings previously observed in threatening conditions, namely, that reward conditioning can also enhance episodic memory generalization, highlighting the interaction of stimulus generalization with the mnemonic process. What is more, this study is the first to compare different effects on the generalization of different reward components. This new body of evidence can be instructive in learning how reward components affect memory trace, boost meaning and gist to facilitate relational processing, or make representations more distinct to reduce interference.

## Figures and Tables

**Table 1:** Rewarded-related predictors across Experiments 1, 2a, and 2b.

Predictor	Description	Expt. 1 Value	Expt. 2a Value	Expt. 2b Value
Expected Value (EV)	Probability of obtaining a reward multiplied by the reward magnitude	5, or 0	5, or 5	8, or 2
Outcome (O)	The predetermined value of the picture that participants could learn from the feedback	10, 0, or 0	10, 0, or 5	10, 0, or 0, 10
Signed Reward Prediction Error (SRPE)	The reward outcome minus the expected value	5, −5 or 0	5, −5, or 0	2, −8, or −2, 8
Unsigned Reward Prediction Error (URPE)	The absolute value of reward prediction error	5, or 0	5, or 0	2, 8, or 2, 8
Uncertainty (U)	The entropy of reward probability $–\left[\begin{array}{c} P*\log\left(P\right)+\\ \left(1-P\right)*\log\left(1-P\right)\; \end{array}\right]$ (the log base is 2)	1, or 0	1, or 0	0.72, or 0.72

**Table 2:** The *M* (*SE*) of old, similar, and new responses were given to the target, lure, and foil items in different conditions in Experiments 1, 2a, and 2b.

Stimulus Type			Targets			Lures			Foils		
Response Type			Old	Sim	New	Old	Sim	New	Old	Sim	New
Exp 1	5 min delay	CS+ *	0.66 (0.03)	0.15 (0.02)	0.19 (0.02)	0.41 (0.03)	0.34 (0.03)	0.25 (0.03)	0.07 (0.02)	0.11 (0.02)	0.82 (0.03)
		CS−	0.56 (0.03)	0.18 (0.03)	0.26 (0.03)	0.39 (0.03)	0.28 (0.03)	0.33 (0.03)	0.11 (0.02)	0.10 (0.02)	0.79 (0.03)
	24 h delay	CS+	0.64 (0.03)	0.15 (0.02)	0.21 (0.02)	0.46 (0.03)	0.24 (0.03)	0.30 (0.03)	0.13 (0.02)	0.08 (0.02)	0.79 (0.03)
		CS−	0.48 (0.03)	0.17 (0.03)	0.35 (0.03)	0.33 (0.03)	0.24 (0.03)	0.44 (0.03)	0.12 (0.02)	0.13 (0.02)	0.75 (0.03)
Expt. 2a (U)	24 h delay	U (1)	0.62 (0.03)	0.18 (0.02)	0.20 (0.02)	0.47 (0.03)	0.26 (0.03)	0.27 (0.02)	0.13 (0.01)	0.12 (0.02)	0.75 (0.03)
		U (0)	0.50 (0.03)	0.17 (0.03)	0.33 (0.03)	0.33 (0.02)	0.25 (0.03)	0.42 (0.03)	0.13 (0.01)	0.14 (0.03)	0.73 (0.03)
Expt. 2b (EV)	24 h delay	EV (8)	0.61 (0.03)	0.17 (0.02)	0.22 (0.03)	0.43 (0.03)	0.26 (0.02)	0.31 (0.03)	0.11 (0.01)	0.09 (0.01)	0.80 (0.02)
		EV (2)	0.52 (0.03)	0.19 (0.02)	0.29 (0.03)	0.33 (0.02)	0.28 (0.03)	0.39 (0.03)	0.09 (0.01)	0.11 (0.02)	0.80 (0.02)
Expt. 2b (URPE)	24 h delay	URPE (8) **	0.59 (0.03)	0.17 (0.02)	0.24 (0.03)	0.36 (0.03)	0.32 (0.03)	0.32 (0.03)			
		URPE (2)	0.56 (0.02)	0.18 (0.01)	0.26 (0.02)	0.39 (0.02)	0.26 (0.02)	0.35 (0.02)			

* In the CS+ category of the 5 min delay test condition of Experiment 1, for example, participants recognized 19.8 trials of 30 old targets as old (0.66), 4.5 trials of 30 old targets as similar (0.15), and 5.7 trials of 30 old targets as new (0.19) on average. Similarly, we can calculate the means of three responses given to three kinds of items in the 24 h delay test condition of Experiment 1, the different uncertainty levels of Experiment 2a, and the different expected value levels of Experiment 2b. ** In particular, for URPE in Experiment 2b, 24 pictures were rewarded with CNY 10 for the high expected value category, higher than the expected value (8), causing a smaller URPE (2) at the reward feedback screen. The remaining six pictures were rewarded with CNY 0, which is lower than this category’s expected value (8), causing a larger URPE (8) at the reward feedback screen. Therefore, within two categories, we could separate 48 trials with low URPE (2) and 12 trials with high URPE (8). For example, in the URPE = 8 condition, participants recognized 28.32 trials of 48 old targets as old (0.59), 8.16 trials of 48 old targets as similar (0.17), and 11.52 trials of 48 old targets as new (0.24) on average. Moreover, because we could not separate different responses given to new foils according to the different URRE levels, the columns that referred to foils with different URPE levels are blank.

**Table 3:** Generalized linear mixed-effects model comparison in Experiment 2a.

Model (M)	Old, Similar, and New Responses to Lures		Old and Similar Response to Lures	
	*BIC*	*BF* _B − M_	*BIC*	*BF* _B − M_
Baseline (b)	3253.60	1.77 × 10^11^	2057.80	984,609.11
U + (1|subject)	3214.00	445.86	2059.50	2,303,637.61
U + (1+U|subject) *	3201.80	1	2030.20	1

* The first column shows the predictors in each model. U + (1+U|subject) represents the regression equation of generalization equal to the fixed effect of the uncertainty and the random effect, which contains an intercept and U as a slope that varies with different individuals. U represents the fixed effect, and random effects are shown in parentheses. U served as random slopes at the individual level in the random effect. One means the random effect of the subject with an intercept. The baseline model only contained the subject as a random effect with no fixed effect. The BIC column shows the BIC value for each model. We calculated the Bayes Factor value for each model compared to the best model in the BF row. The best model has the lowest BIC values and a BF value of 1.

**Table 4:** Generalized linear mixed-effects model comparison in Experiment 2b.

Model (M)	Old, Similar, and New Responses to Lures		Old and Similar Response to Lures	
	*BIC*	*BF* _B − M_	*BIC*	*BF* _B − M_
Baseline(b)	3974.10	34,012,706,080	2585.40	417,585,553.60
EV + URPE + (1|subject)	3953.50	1,143,952.58	2576.80	5,666,034.23
EV + URPE + (1+EV|subject)	3930.20	9.97	2545.70	1
EV + URPE + (1+EV|subject) + (1+URPE|subject) *	3951.50	420,836.64	2567.1	44,355.85
EV + (1|subject)	3948.70	103,777.04	2579.70	24,154,952.75
EV + (1+ EV |subject)	3925.60	1	2549.30	6.05

* The maximal model (generalization~EV + URPE + (1+EV|subject) + (1+URPE|subject)) failed to converge at first. We used the BOBYQA optimizer and increased the maximum number of iterations to 10,000 to converge it successfully.

## Data Availability

The data for all experiments are available at https://osf.io/mbxjg (accessed on 28 October 2022).
